# Defending and Defining Environmental Responsibilities for the Health Research Sector

**DOI:** 10.1007/s11948-024-00487-z

**Published:** 2024-06-06

**Authors:** Bridget Pratt

**Affiliations:** Queensland Bioethics Centre, Australian Catholic University, Brisbane, Australia

**Keywords:** Ethics, Health research, Justice, Environment, Climate change

## Abstract

Six planetary boundaries have already been exceeded, including climate change, loss of biodiversity, chemical pollution, and land-system change. The health research sector contributes to the environmental crisis we are facing, though to a lesser extent than healthcare or agriculture sectors. It could take steps to reduce its environmental impact but generally has not done so, even as the planetary emergency worsens. So far, the normative case for why the health research sector should rectify that failure has not been made. This paper argues strong philosophical grounds, derived from theories of health and social justice, exist to support the claim that the sector has a duty to avoid or minimise causing or contributing to ecological harms that threaten human health or worsen health inequity. The paper next develops ideas about the duty’s content, explaining why it should entail more than reducing carbon emissions, and considers what limits might be placed on the duty.

## Introduction

We are in the midst of an environmental crisis that will only get worse, unless significant changes are rapidly made. So far, *six* of the nine planetary boundaries have been exceeded, including climate change, loss of biodiversity, chemical pollution, freshwater use, and land-system change (Richardson et al., 2023). Once these thresholds are crossed, it could generate unacceptable environmental change, often with deleterious or potentially even disastrous consequences for humans and nonhumans (Rockström et al., 2009). For example, as temperatures worldwide continue to rise, we are already seeing more frequent heatwaves and bushfires, greater frequency and intensity of heavy precipitation events, increased risk of drought and reduced water availability, sea level rise, and further increases in ocean temperatures and acidification (IPCC 2022a, 2022b, 2018). These effects impact those already experiencing marginalisation and disadvantage most, widening disparities in human health and wellbeing between and within countries (Harrold et al., 2012). They destroy the natural habitats and ecosystems that most (if not all) species rely on for food, shelter, and other vital resources. In effect, we are seeing and will continue to see the loss of animal and plant species.

There is increasing evidence that healthcare systems are contributing to the environmental crisis we are facing. They produce large amounts of greenhouse gas emissions, hazardous and non-hazardous waste, and air pollutants (Lenzen et al., 2020; Eckelman & Sherman, 2016; NHS, 2020). On average, they are responsible for 5.2% of worldwide carbon emissions (Romanello et al., 2022). Healthcare systems also affect aspects of circularity and biodiversity via their material extraction, blue water consumption, and land use practices (Steenmeijer et al., 2022). They are thus contributors to our exceeding the six planetary boundaries.

In contrast, few studies quantify the environmental impact of conducting *health research* (Billiones, 2022). Those that do primarily assess the *carbon emissions* of clinical or pragmatic trials. So far, no studies have sought to measure other environmental impacts of health research. In 2007, the Sustainable Trials Study Group was the first to measure the carbon emissions of a multicentre international clinical trial. Total carbon emissions for the CRASH-1 trial were estimated at 629 tonnes (Sustainable Trials Study Group, 2007). Most recently, Adshead and colleagues estimated that 27.5 million tonnes of emission gases are attributable to clinical trials globally (Adshead et al., 2021). Others estimate the aggregate global emissions of the pharmaceutical sector to be about 52 million tonnes (Belkhir & Elmeligi, 2019).

The collective carbon emissions of health research, while sizeable, are thus likely much lower than the total emissions of healthcare systems globally. In fact, they seem roughly on par or slightly above the emissions of single healthcare systems like Australia (35 million tonnes) or Canada (33 million tonnes) (Eckelman et al., 2018; Malik et al., 2018). In most countries, the healthcare sector’s carbon footprint is comparable in size to the food sector. It is only surpassed by the energy, transport, agriculture, and construction sectors (Pichler et al., 2019; Eckelman et al., 2018). For example, in Australia, the annual carbon emissions of healthcare are about half those of the construction sector (Malik et al., 2018). In effect, the total emissions of health research would also fall below those of food, agriculture, energy, transport, and construction sectors.

Even so, Subaiya and colleagues (2011) demonstrated that clinical trials’ carbon emissions can be substantially reduced by using strategies outlined in the UK National Institute of Health Research’s *Carbon Reduction Guidelines*. When the CRASH-2 trial used several of those strategies, its total annual emissions were 73 tonnes less than the CRASH-1 trial (Subaiya et al., 2011). Lyle and colleagues (2009) further affirm that emissions from pragmatic randomised controlled trials are generated in areas where steps could be taken to reduce them. Most existing laboratories could reduce their energy consumption by 30 to 50% using existing technology (Lopez et al., 2017). Concerningly, however, the health research sector has done very little in the past ten years to reduce its emissions (Adshead et al., 2021), even as the urgency of the environmental crisis has greatly increased.

While health research could reduce its emissions and perhaps its other environmental impacts, the normative question raised is: should the sector do so given it is a relatively low emitting sector whose other ecological harms are less clear? Whether philosophical or ethical grounds exist to support the claims that health research should minimise its climate change impact or its overall environmental impact have not been investigated by the research ethics community. This reflects that modern bioethics and environmental ethics have historically evolved as distinct and disparate fields (Lee, 2017). To some extent, that separation is starting to be bridged. Growing recognition of the deep intersection between human health and the environment has meant scholars increasingly advocate for readopting a broader perspective in bioethics that considers the natural environment (Lee, 2017; Resnik, 2009; Dwyer, 2009). Their work primarily connects environmental ethics to public health ethics (see Lee, 2017; Timmermann et al., 2022) and clinical ethics (see Macpherson, 2014; Richie, 2019, 2022; Thiel & Richie, 2022).

So far, very limited effort has been made to forge connections between research ethics and environmental ethics. At most, several specific topics like recombinant DNA techniques, dual use research, and the CRISPR method of gene editing link research ethics to consideration of harms to nature (Hurlbut et al., 2015; Caplan et al., 2015; WHO, 2022). Initial ethics discourse on genetics research largely focused on the worry that dangerous material might result from recombinant DNA research and escape into the environment (Selgelid, 2007). While virtually all life sciences research has dual-use potential, concern has been specifically raised in relation to “research that, based on current understanding, can be reasonably anticipated to provide knowledge, products, or technologies that could be directly misapplied by others to pose a threat to public health and safety, agricultural crops and other plants, animals, the environment, or material” (Rath, Ishi, & Perkins, 2014). The CRISPR method comes with the possibility of creating edited insects or animals that prove harmful to other organisms, the environment, and/or humans (Caplan et al., 2015).

As part of the movement to readopt a broader perspective in bioethics, Samuel and Richie (2022) recently argued that we need to go further and rethink the *entire* research ethics paradigm so that research ethics concepts, responsibilities, and frameworks include notions of environmental sustainability. Their rationale is that conceptions of harm and benefits in health research are anthropocentric. Evidence that health research generates carbon emissions, which contribute to climate change and biodiversity loss, thus justifies rethinking the research ethics paradigm to account for harms (and benefits) to nature (Samuel & Richie, 2022). Yet, as I have just laid out, existing evidence shows those harms may not be so sizeable. Before rethinking the entire paradigm, we need a deeper interrogation of why the health research sector should minimise its overall environmental impact (and not just its climate change impact).

In this article, I argue that strong philosophical grounds, derived from theories of health and social justice, exist to support the claim that health research should avoid or minimise causing or contributing to harms to nature—ecological harms—that push humans below adequate health, especially those who are systematically disadvantaged. I then consider what upholding that duty should entail by assessing to which ecological harms the health research sector contributes. To identify ecological harms, I draw on ecological justice theory, which characterises human harms to the environment that matter from an ethical perspective and should be avoided.^1^ Those ecological harms clearly relate to and help push us towards the planetary boundaries discussed earlier, including but not limited to climate change. To conclude, I articulate what limits might be placed on the duty. Although it is beyond the scope of the paper to specify environmental responsibilities for particular health research actors (e.g., governments, funders, research institutions, researchers, journals, research ethics committees) in the public and private sectors, this is recognised as is an important avenue of future investigation. In this paper, the terms nature, the environment, and nonhumans are used interchangeably to refer to living beings (e.g., animals and plants), non-living entities (e.g., water, soil), and collectives (e.g., species, ecosystems).

## Grounds for Health Research to Minimise its Environmental Impact

Theories of social justice describe what is required of social institutions in just societies. Rawls argued that basic structure institutions should be subject to principles of justice because of the important role they play in preserving background justice and the effects such structures have on those who live within them. Wenner (2018) contends that the clinical research enterprise is akin to other institutions commonly included under the rubric of the basic structure in that the life prospects of all people are unavoidably and profoundly affected by it. Clinical research plays a pivotal role generating the knowledge underlying the “health systems that are available to individuals, that individuals cannot opt out of, and that will have deep and lasting impacts on their life prospects, their final ends and purposes, and the way that they think of themselves” (Wenner, 2018, p. 31). While Wenner’s focus is on clinical research, I submit that her reasoning extends to health research more broadly. Basic science, genomics, and health services research also play a pivotal role generating the knowledge underlying our health systems, and public health research fulfils a comparable role for our public health systems. Philosophers Alex London and Jennifer Ruger similarly identify health research as a basic social institution (London, 2022; Ruger, 2010).

Theories of health and social justice purport that, within a just state, its social institutions must secure the basic interests of its members, including their health and wellbeing (Nussbaum, 2006; Powers & Faden, 2006; Venkatapuram, 2011). This requires social institutions to secure an adequate^2^ level of health for all members of society, independent of gender, ethnicity, sexual orientation, place of birth or residence, social status, political beliefs and religion (Powers & Faden, 2006; Ruger, 2010; Pogge, 2002). As people cannot opt out of basic social institutions, anything less would create vast health inequities. Multiple theories further purport that priority should be given to bringing those individuals, groups, and communities who are considered systematically disadvantaged^3^ up to an adequate level of health (Powers & Faden, 2006; Wolf & de Shalit, 2007). In effect, social institutions must ensure equal access to high-quality public health measures (Powers & Faden, 2006; Daniels, 2008; Ruger, 2010), high-quality healthcare and services (Daniels, 2008; Ruger, 2010), and the social determinants of health, especially for the worst-off (Daniels, 2008; Venkatapuram, 2011). These requirements primarily apply to basic social institutions with an explicit health focus—namely, public health, healthcare, health research—and to those basic social institutions that impact the social determinants of health (Daniels, 2008; Venkatapuram, 2011).

According to theories of health and social justice, the primary duty of the health research sector is then to help secure an adequate level of health for all members of society, with some priority given to those who are considered systematically disadvantaged. The sector can achieve that mission by generating knowledge about the causes of poor health, especially for those considered systematically disadvantaged; about effective public health interventions, healthcare and services, and measures to ensure the social determinants of health, particularly those needed to achieve adequate health for the worst-off; and about how to organise social institutions (including but not limited to the public health and healthcare systems) to ensure equal access, especially for those least advantaged. Similarly, London (2022) affirms that, as a matter of social justice, it is a duty of the health research sector to close knowledge gaps to enable healthcare systems to avoid premature death and severe morbidity and to reduce and eliminate disparities in the ability of healthcare systems to meet people’s needs. Although he does not link health research’s knowledge contribution to the public health system or to basic social institutions that impact the social determinants of health, doing so follows directly from social justice theory. Helping secure adequate health requires a knowledge base for our public health, healthcare, and broader social system. This encompasses generating some knowledge related to the environment such as information about environmental causes of poor health, including the negative health effects caused by the environmental footprint of health research and other sectors, and about effective ways the healthcare sector can adapt to the environmental crisis.

However, the health research sector’s primary duty is countered where it generates ecological harms that threaten human health and health equity in its pursuit of that knowledge. Increasing evidence exists that altering our planet’s climate contributes to worsening population health and widening health disparities. Human health impacts of climate change are already being observed and are predicted to worsen—namely, increasing chronic undernutrition, water scarcity, food insecurity, respiratory impacts (e.g., chronic obstructive pulmonary disease, asthma), vector-borne and waterborne illnesses, and heat stroke and death (Watts et al., 2018; Zhao et al., 2021). Those already experiencing marginalisation and disadvantage are the most severely affected (IPCC, 2018). Beyond global warming, human activities generate other effects or products that damage the environment, pushing us farther towards or past planetary boundaries and threatening human health. They include hazardous waste, biodiversity loss, and air pollution (Sala et al., 2009; Manisalidis et al., 2020; Myers et al., 2013). How health research can cause or contribute to ecological harms beyond greenhouse gas emissions and climate change is discussed further in the next section.

Ultimately, to advance health and social justice, the health research enterprise must minimise its environmental impact. It should avoid causing or contributing to ecological harms that push humans (farther) below adequate health, especially those who are already systematically disadvantaged. As the sector comprises a basic social institution from which individuals cannot opt out, its effects cannot be avoided by members of a given society. Upholding the duty is then essential to ensure adequate health for them. Beyond health and social justice, doing so also advances environmental justice on two dimensions: wellbeing and distribution. Environmental justice calls for fairly distributing environmental benefits and harms and for avoiding and addressing harmful effects of environmental degradation on the ability of humans to develop and function (Schlosberg & Carruthers, 2010; Schlosberg, 2007). Avoiding ecological harms that threaten human health helps ensure the environmental conditions necessary for human wellbeing and flourishing. Avoiding ecological harms that threaten health equity helps ensure that the health effects of environmental damage are not disproportionately distributed to the systematically disadvantaged.

Objections could be raised that the health research sector does not have a duty to minimise its environmental impact as a matter of social justice because the claim of no difference applies. Relative to other sectors like healthcare or agriculture, it is possible that health research’s greenhouse gas emissions and other ecological harms generate a negligible amount of impact and reducing them won’t make a meaningful difference to mitigating the environmental crisis. The claim of no difference has previously been raised to argue that individuals do not have an obligation to reduce their carbon emissions (Johnson, 2003; Sandberg, 2011; Sinnott-Armstrong, 2005). It could potentially apply to the health research sector as well because, so far, initial data puts total clinical trial emissions well below other sectors’ emissions. More applied types of health research may well generate very small emissions and other environmental impacts.

Yet, even if the health research sector is proven not to generate substantial emissions or other ecological harms, I establish below that it meets Cripps’ criteria for weak collective responsibility if they are extended from individual persons to individual sectors or industries (Cripps, 2011). Cripps’ criteria, adapted for sectors, would identify weak collective responsibility where:

- the sectors acted in ways which, in aggregate, caused harm, and which they were aware (or could reasonably be expected to have foreseen) would, in aggregate, cause harm (although each only intentionally performed its own act);
- they were all aware (or could reasonably be expected to have foreseen) that there were enough others similarly placed (and so similarly motivated to act) for the combined actions to bring about the harm; and.
- the harm was collectively avoidable: by acting otherwise (which it/they could reasonably have done), the sectors could have avoided the harm.

By not reducing its greenhouse gas emissions, the health research sector is contributing to aggregate harm. Given the links between greenhouse gas emissions and climate change are well known, the sector could reasonably be expected to have foreseen the impact of its actions. As the next section demonstrates, the healthcare sector can cause or contribute to other ecological harms which, in aggregate, are detrimental to climate change and additional planetary boundaries: land system change, rate of biodiversity loss, and chemical pollution. The links between those harms and the planetary boundaries are clear, so the sector could reasonably be expected to have foreseen them too. It is also reasonable to think that the sector could have minimised its greenhouse gas emissions and other ecological harms and, thereby helped avoid their aggregate impact. Evidence exists that doing so is feasible (Subaiya et al., 2011; Lopez et al., 2017).

A duty thus exists, despite health research not being proven to generate substantial emissions, because it meets the criteria for weak collective responsibility. In cases of weak collective responsibility, the primary corresponding duty is naturally a collective one: to do something about the harm together. That is, to act, qua group, to bring about an end to the harm (Cripps, 2011). All sectors that meet the criteria for weak collective responsibility, including the health research sector, are responsible for minimising their environmental impact.

## The Duty to Minimise Health Research’s Environmental Impact

If we assume that the health research enterprise has a duty to avoid or minimise causing or contributing to ecological harms that worsen human health and/or widen health inequities, what does that duty entail? The duty’s content depends on the particular ecological harms to which health research contributes. As such, we need to determine what types of ecological harms are generated or exacerbated by health research and which of those specific harms negatively affect human health. This section, therefore, develops ideas about the duty’s content by exploring three key questions: (1) what types of ecological harms exist, (2) is health research likely to cause or contribute to those harms, and (3) if so, do they push humans (farther) below adequate health, including those who are already systematically disadvantaged. To consider the first question, I draw on ecological justice theory, which characterises different ways human activities can harm nature. Those harms should be avoided because they comprise injustices to nature or unfairness in our treatment of nature. They encompass not only the greenhouse gas emissions causing climate change but also other ecological harms that contribute to climate change and/or push us towards/past additional planetary boundaries like biodiversity loss, land-system change, and fresh water.

Ecological justice theory purports that injustice to nature can occur across several different interrelated dimensions: functioning, power, harmony, and recognition. The functioning dimension calls for ensuring nature’s ability to function and finds harm—injustice—in forces that limit that potential (Schlosberg, 2019). Human activities harm nature where they adversely impact or thwart the living process or interrupt the life project or telos^4^ of nonhumans (Schlosberg, 2019). That occurs when humans directly destroy nature or where they generate certain products like hazardous waste, excessive non-hazardous waste, greenhouse gas emissions, and/or air pollutants (Capon et al., 2020). Below, I describe what features of health research have such effects and thereby cause or contribute to interrupting nature’s functioning.

Certain features of health research give rise to greenhouse gas emissions. Studies show that clinical and pragmatic trials’ carbon emissions are generated by the following four components:

1. Building energy use.
2. Trial team travel: commuting, international travel.
3. Participant travel: commuting to and from the trial site.
4. Procurement: distribution and delivery of equipment, reagents, and medical products (Lyle et al., 2009).

These features are common to other types of health research, which means they too will (to varying extents) generate carbon emissions and contribute to global warming. For example, basic science research has a higher level of energy consumption. Laboratories typically consume three to six times more energy per square foot than do office buildings, with much of that due to refrigeration and ventilation systems (Lopez et al., 2017; Madhusoodanan, 2020).

What research is designed to do and how it is designed can also negatively affect nature’s functioning by generating emissions or directly destroying nature. As an example, gene editing studies that create genetically modified organisms have the potential to generate products—edited insects or animals—that interrupt the living process or telos of other nonhumans (Caplan et al., 2015). Depending on how research is designed, it can arguably generate higher or lower greenhouse gas emissions. Low carbon models of healthcare delivery entail using low carbon information, communication, and technology (ICT) pathways; using green medicines (wherever possible) and green medical technologies (i.e., ones with lower environmental impact); eliminating redundant tests; and eliminating certain anaesthetic gases (Schroeder et al., 2013). Different types of health research will differ in their use of ICTs, medicines, medical technologies, and anaesthetic gases, and in the extent to which they run tests on patients. For example, big data research and decentralised clinical trials are very ICT intensive (Eugene, 2022; RAND, 2023). Specific low carbon features will thus vary between types of health research. Even so, the bottom line is that, where models of research have low emissions features, their emissions will be reduced. Where they do not, they can potentially generate substantial emissions.

Beyond its building energy use, travel, procurement, and research design, health research can negatively affect nature’s functioning by generating hazardous waste, non-hazardous waste, or both (Janik-Karpinska et al., 2023; Padmanabhan & Barik, 2019). Basic science and clinical research, for example, involve significant utilisation of single-use products (e.g., pipette tips, gloves) and create a sizeable amount of plastic waste (Madhusoodanan, 2020; Bell, 2019; Mesa, 2022; Urbina et al., 2015).^5^ 15% of the waste generated by healthcare activities, including research, is hazardous (i.e., infectious, toxic, chemical, radioactive) (Janik-Karpinska et al., 2023).^6^ Where health research involves antibiotics, for instance, it can generate chemical waste that harms the environment (Polianciuc et al., 2020).^7^

Finally, disposal of hazardous and non-hazardous waste can generate products that destroy nature. Depending on how disposal is achieved, it can cause air pollution, water and soil contamination, and greenhouse gas emissions due to the gases and chemicals evaporating from the waste (UN Environment Program; WHO, 2018). Several features of health research—building energy use, travel, procurement, research design, waste generation and disposal—then cause or contribute to interrupting nature’s functioning.

When health research interrupts nature’s functioning, it contributes to ecological harms that can worsen human health and/or widen health inequities. Greenhouse gas emissions, air pollution, and waste contribute to global warming and climate change, which, as previously noted, disproportionately burdens the poor and least advantaged (Harrold et al., 2012). Impacts of plastic components on human health include endocrine disruption, psychological and neurological effects, and impairment of liver and kidney function (Cook & Halden, 2020). Hazardous waste exposure and chronic air pollution directly increase the risk of respiratory illnesses, cardiovascular disease, and cancer. Some hazardous substances can also cause genetic mutations, physiological malfunctions (e.g., reproductive impairment, kidney failure, etc.), and birth defects (EPA, 2023). The clearest consequence of antibiotic release (hazardous chemical waste) in natural environments is the selection of resistant bacteria. That has significant implications for human health because it reduces the effectiveness of antibiotics in treating infectious diseases (Martinez, 2009).

The next harm identified by ecological justice theory is *extractivism*. The power dimension of ecological justice finds injustice where unfair power dynamics between humans and nature are reinforced. That occurs where nature is treated as a resource for unlimited human exploitation and used as a mere means to an end (Boff, 1997). Extractivism is characterised by over-exploitation (Kelbessa, 2014) of and violence (Ulloa, 2017; Whyte, 2018) to nature. Human activities harvest a species or entity at rates that cannot be compensated for by reproduction, regrowth, or recharge (Maxwell et al., 2016). The health research sector can cause or contribute to extractivism where it makes excessive use of water locally or worsens existing scarcity. As an example, laboratories are significant consumers of water (Lopez et al., 2017). While autoclaving avoids the ecological harms that medical incineration can cause, steam autoclaves are a major source of laboratory water consumption. The sterilization of equipment cannot happen without water to generate the steam (Mechler, 2023).

The health research sector may further contribute to extractivism through the medical products and equipment it utilises. That can occur where the *materials* being used to create them are excessively used by the sector or are already scarce locally, nationally, and/or globally. Healthcare services rely on an enormous array of natural resources, including common and rare metals, rubber, petroleum, and biomass (Jameton & Pierce, 2001). Health research would similarly depend on many of these natural resources where it involves using medical products and equipment or carrying out laboratory work. For instance, basic science research and clinical trials rely on rubber in their use of latex gloves and, in doing so, contribute to its overexploitation. The global supply of natural rubber is currently under threat (Swain, 2021).

Extractivism has severe impacts on human health that vary depending on the particular resource being exploited. For example, water insecurity and scarcity increase the likelihood of water and hygiene-related illnesses (Schrecker et al., 2018). Rubber finds many applications beyond health research that are relevant to human health. It is used in condoms and has been particularly important during the Covid-19 pandemic in personal protective equipment for health workers (Swain, 2021). Its scarcity then has implications for family planning and the transmission of infectious diseases. In effect, where health research uses scarce natural resources like rubber or water, it contributes to ecological harms that worsen human health.

Another harm to nature identified by ecological justice theory is *misrecognition*: disrespecting and/or not protecting nonhuman difference and diversity (Whyte, 2018; Rozzi et al., 2015; McGregor, 2009). Injustice thus occurs where biodiversity is not valued and/or lost. Where the health research sector expands existing facilities or builds new research facilities, it can contribute to biodiversity loss. This occurs where land is cleared for facilities’ construction and native biota are removed and/or replaced with cosmopolitan species. Additionally, depending on the natural resources used to make the medical products and equipment studies utilise, it is possible that biodiversity loss could be caused or contributed to by the sector. Certain types of studies further have the potential to generate such effects. An example would be applying CRISPR technology to potentially eradicate a disease by eradicating disease vectors and invasive species. Caplan et al. (2015, p. 1422) note that the use of such gene drives have the potential to “decimate an entire species.”

There are clear connections between biodiversity loss and worsening human health. Biodiversity loss negatively affects food production and mental health and widens the spread of infectious diseases (Sala et al., 2009). Disease ecologists have shown that Lyme disease, Chagas disease, and hantavirus exposure increases with falling mammalian diversity (Myers et al., 2013). But should we conclude that, where health research contributes to biodiversity loss, it *always* negatively impacts human health? It could be argued that eradicating a disease-causing species using CRISPR may actually be good for and not harmful to human health. In response, I affirm, in some cases, that could be true but only where a species’ loss has minimal effect on ecosystem functioning. The loss of any species that fulfil critical roles that no or few other species can fulfil would, in contrast, have a detrimental effect on an ecosystem, which, in turn, would negatively impact human health (Bland, 2016; Bates, 2016). Accordingly, it is most accurate to conclude that, where health research fails to protect biodiversity in its land use, resource use, and/or research design, it often (but not always) contributes to ecological harms that worsen human health.

Finally, ecological justice also finds harm—injustice—where human forces upset *harmony* by interfering with or impairing relationships between humans and nonhumans or between nonhumans (Whyte & Cuomo, 2017; Whyte, 2018; McGregor, 2018). Such relations are disrupted by human actions that change land use patterns, introduce new species that upset local ecosystems, alter nonhumans’ behaviours and interactions, and/or destroy nonhuman habitats. The health research sector can cause those effects via its generation of plastic waste and chemical waste, where it expands existing facilities, and where it builds new research facilities. Here, site selection of new or expanded facilities is an important consideration. Facilities will alter land use patterns and destroy the habitats of plants and animals when untouched land, nature reserves, or parklands are chosen. New facilities or expansions may further be designed with green spaces that introduce new plant species that disrupt the local ecosystem. Plastic waste can also cause habitat destruction/contamination (Cook & Halden, 2020), and chemical pollutants can elicit a range of sublethal effects on animals, even at minute concentrations, that include disrupting their individual behaviour and interactions (Michelangeli et al., 2022). Neurotoxic chemicals, for example, are suspected to produce changes in organism mating behaviour, predator escape response, and feeding behaviours (Legradi et al., 2018). They may also influence collective behaviour, altering collective decision-making, coordination, and overall performance of animal groups (Michelangeli et al., 2022).

Some (but not all) changes in land use can worsen human health by increasing disease risk and prevalence. Where land use changes alter human–wildlife interactions, they can be an important source of zoonotic disease (Myers et al., 2013). The destruction of nonhuman habitats drives animals into new environments and is often followed by urbanization and human population growth. Both bring people into greater direct contact with a wider range of wildlife, which also creates the conditions for new zoonoses to emerge (Vidal, 2020). Changes to food chains, mating behaviours, and predator-prey interactions can potentially have severe ecosystem impacts (Michelangeli et al., 2022; Legradi et al., 2018). Humans need a full environment, including nonsentient life and ecosystem relations, in order to flourish (Schlosberg, 2007). As such, any severe ecosystem impacts would undoubtedly be detrimental to human health. In effect, where health research disrupts interactions between nonhumans through its waste generation and/or land use, it often (but not always) contributes to ecological harms that worsen human health.

To summarise, the four dimensions of ecological justice each capture certain ecological harms. I have shown that health research causes or contributes to those ecological harms and that such harms, in turn, are linked to negatively impacting human health some if not all the time (Table 1). By identifying the types of ecological harm the health research sector is likely to generate and assessing which of them push humans (farther) below adequate health (Table 1), we now have a clearer idea about what the sector’s duty to minimise its environmental impact should entail. It arguably requires avoiding or minimising not only the production of greenhouse gas emissions but also the generation of hazardous waste, plastic waste, and air pollutants; excessive use of particular resources; use of particular scarce resources; most biodiversity loss; habitat destruction; and some land use changes. Upholding the duty will hinge on the following components of the research enterprise: travel, building design and energy use, water use, waste generation and disposal, research design, equipment and medical products used, and site selection for research infrastructure/facilities.

**Table 1:** Ecological harms caused or contributed to by health research and their effects on human health

Dimension of ecological justice	Associated ecological harm(s) linked to health research	Harmful to human health
Functioning	- Air pollution - Waste generation - Greenhouse gas emissions	Yes Yes Yes
Power	Overexploiting resources	Sometimes
Recognition	Biodiversity loss	Sometimes
Harmony	- Land use change - Introduce new species - Habitat destruction - Waste generation	Sometimes Sometimes Yes Sometimes

## Limits to Upholding the Duty

This section considers what limits should ideally be placed on the health research sector’s duty to avoid or minimise ecological harms that worsen human health and health equity and what limits might be placed on the duty based on its grounding in social justice theory. An important concern is that minimising the health research sector’s environmental impact will compromise its capacity to generate knowledge to help ensure adequate human health and to help reduce health disparities. As discussed previously, generating such knowledge is the health research sector’s primary duty as a matter of social justice. For some health research projects, minimising ecological harms could plausibly reduce or compromise their scientific validity in ways that cannot be sufficiently mitigated. Within the healthcare sector, there is concern that measures to minimise its environmental impact can, at times, conflict with delivering the best patient care (Quitmann et al., 2023). For instance, medical imaging may be limited for patients if energy consumption has to be considered (Quitmann et al., 2023). Similarly, certain elements of research projects needed for scientific rigor may be energy-intensive or otherwise environmentally harmful. They could be limited in studies if environmental impact has to be considered.

Yet favouring minimising ecological harms in contexts where doing so compromises scientific validity will not necessarily *always* interfere with upholding the sector’s primary duty as a basic social institution. Whether it does so depends on the social value of a given study. Most existing accounts hold that health research is socially valuable when it uses sound scientific methods in order to generate information that can be used to protect or promote the health of individuals and populations (Rid, 2020). On that view, judging the social value of health-related research requires considering ex-ante how much the knowledge it generates can help to improve human health and how likely the resulting health improvements are to materialize (Rid, 2020). For studies with a high magnitude and likelihood of generating health benefits but whose scientific validity would be unavoidably compromised by minimising ecological harms, the two duties will conflict. Favouring the duty to minimise ecological harms will come at the expense of the health research sector’s primary duty.

A limit to the duty to minimise ecological harms should then arguably occur in most, *but not all*, circumstances where studies meet the two criteria of unavoidably compromised scientific validity and high social value. In circumstances where the extent of harm to the environment and (indirectly) to human health is sufficiently severe, it *may* ethically justify not permitting such studies to be conducted at all or in a particular location.^8^ That is because the social value of a health research project should outweigh the risks involved (Rid, 2020). I acknowledge that the given level of risk studies’ social value should be sufficient to justify is typically thought to be the risk to *participants* (Wendler & Rid, 2017). The rationale is that study participants are used as a means of conducting the research and achieving whatever good should thus justify putting them at risk (Walen, 2020). In circumstances where studies cause severe ecological harms, the risk is not directly to participants but to nature and indirectly to human participants’ and bystanders’ health. Bystanders are individuals or groups who are not participants in health research but who are nevertheless exposed to burdens as a result of a study’s conduct (Kimmelman, 2007).

Like human participants, the environment is used as a means in health research projects. Energy, animals, water, and other nonhumans are all used in the service of conducting health research. As such, achieving whatever good should justify destroying or harming them. Bystanders, on the other hand, are not used as a means in health research projects. Even so, several bioethicists have explored the limits of acceptable risk for research bystanders (Shah et al., 2018; Jamrozik & Selgelid, 2021). Shah et al. (2018) affirm it is important to protect all research bystanders where they may be unable to protect themselves. Where health research generates ecological harms, its impact on participants' and bystanders’ health will likely be felt most by those who are already worst-off and unable to protect themselves. They are exposed to persistent, intersecting, entrenched structural inequities, making them particularly vulnerable to harm from, for example, the hazards unleashed by climate change (Dunlap & Brulle, 2015). If we accept that studies’ social value is pertinent to consider in relation to risk and that encompasses risks to participants, bystanders who cannot protect themselves, and nature, then severe harms to nature and indirectly to the health of participants and bystanders could outweigh the social value of certain health research studies.

I now briefly turn to two key ways the scope of the health research sector’s duty to minimise its environmental impact could be restricted based on its grounding in social justice theory. First, as social justice theories are typically state-based, it could be argued that the health research sector’s duty is limited to the nation-state. A given health research sector is only responsible for minimising ecological harms that affect the health and wellbeing of people within its own country. In effect, externally-funded health research only needs to minimise ecological harms in host countries if those harms affect people in the country funding the study. Yet, externally-funded health research is often funded by high-income countries and performed in low- and middle-income countries, where those most affected by the impacts of climate change largely reside (Harrold et al., 2012). Generating ecological harms in these countries would only burden their populations more and worsen their health further.

This concern takes us back to debates on the scope of justice: do we owe obligations of justice globally or only to those within our nation-state? Whether or not the duty to minimise ecological harm extends beyond the nation-state depends on whether or not the obligation not to harm extends globally. That matter is the focus of much scholarship on global justice theory.^9^ Future work could, therefore, usefully consider global justice theories, spanning different stances on the scope of justice, and assess what duties (if any) they ground to members of other societies in terms of health research and environment. Solidarity theory, reparations theory, and climate justice theory may also be pertinent to apply in that work as well.

Second, concern could be raised that the duty focuses on minimising ecological harms that worsen human health and health equity but ignores minimising ecological harms that erase cultural diversity or reinforce unfair power dynamics between humans, except where those harms generate negative health effects too. This too stems from grounding the duty in social justice theory as opposed to environmental justice theory. As previously discussed, minimising ecological harms that worsen human health and health equity advance the wellbeing and distributive dimensions of environmental justice. But they do not advance its recognition or power dimensions. The former calls for respecting and protecting the environmental identities and heritages of different peoples and their local or traditional environmental/ecological knowledge (Figueroa, 2006; Whyte, 2017; Whyte, 2018). The latter requires reducing unfair power dynamics between humans that involve nature and its degradation. For instance, damaging the lands of Indigenous peoples reinforces coloniality (Whyte, 2016).

This limitation is morally concerning because, if the health research sector generates ecological harms that contribute to the erasure of place-based cultures and/or reinforce unfair power dynamics that involve nature, it violates environmental justice. Future research could thus usefully explore whether theories of environmental justice establish duties for health research and, if so, what their content is. At the same time, this concern does not reject my claim that the research sector has a duty to minimise its environmental impact. Instead, it suggests the duty grounded in this paper does not perhaps go far enough.

## Conclusions

This paper argues that health research has a duty to minimise its environmental impact as a matter of health and social justice. Even though the health research sector has not been proven (so far) to generate substantial ecological harms, the duty applies because the sector meets the criteria for weak collective responsibility. Based on what types of ecological harms are likely linked to the health research enterprise and that negatively affect human health and health equity, upholding the duty requires avoiding or minimising not only the production of greenhouse gas emissions but also the generation of hazardous and non-hazardous waste and air pollutants; use of particular scarce resources; excessive use of particular resources; most biodiversity loss; habitat destruction; and some land use changes. Limits to the duty may exist for studies with high social value, where their scientific validity is unavoidably compromised by minimising certain ecological harms and those ecological harms are not severe.

Once we view minimising ecological harm as a requirement of health and social justice for basic social institutions, the moral importance of broadening research ethics to consider the environment is also further clarified. Rethinking the research ethics paradigm effectively promotes health and social justice. Going forward, it is thus imperative to build further connections between research ethics, environmental ethics and philosophy, and the planetary sciences. We need more discussion and consideration within the field of research ethics about how the health research enterprise should minimise its environmental impact and who is responsible for doing what to achieve it; whether upholding the duty generates tensions with other duties held by health research sector and how best to navigate those tensions; and how those answers should inform the revision of existing concepts in research ethics like balancing risks and benefits, minimal risk, fair benefits, and compensation for research-related harms.

Although this paper focused on how health research can harm the environment, we must also recognise that health research can help protect the environment. For instance, health research can generate information on how healthcare sectors can mitigate their environmental impact (see Thiel et al., 2018). That health research can generate knowledge that benefits the environment has implications for concepts in research ethics such as social value, balancing risks and benefits, and fair benefits. Future work should explore whether grounds exist to reimagine such concepts to take account of health research’s potential to generate positive environmental impacts and, if so, how to revise them. Revised concepts should then be incorporated into research ethics guidelines and frameworks. At present, research ethics guidelines and frameworks do not consider the environmental impact—negative or positive—of health research. Even ethics frameworks linking research to social justice—London’s research for the common good and Pratt’s research for health justice—fail to do so (see Pratt, 2021; London, 2022). Rectifying this state of affairs should be viewed as a priority for immediate action by the field.

## Footnotes

1 In contrast, environmental justice, and the related concept of climate justice, focus on the harmful and unequal effects of environmental degradation and climate change on the ability of humans to develop and function (Low & Gleeson, 1998; Moellendorf, 2015). They thus characterise harms to humans that involve nature.

2 Debate continues as to whether adequate health comprises a basic, sufficient, or optimal level (see Shue, 1996; Pogge, 2002; Powers & Faden, 2006; Ruger, 2010).

3 Systematic disadvantage means individuals, groups, or communities experience deficits below sufficiency on multiple dimensions of wellbeing. Dimensions of wellbeing include health, reasoning, personal security, respect, and affiliation (i.e., relations of love, friendship) (Powers & Faden, 2006).

4 The telos for being refers to the specific role that a non-living entity (e.g., soil) or collective (e.g., ecosystem) plays in the environment.

5 Plastic waste poses a spectrum of risks to wildlife. It is the main cause of entanglement of animals and marine life, resulting in their injury and mortality. Its ingestion adversely impacts organisms’ fitness and functions, such as food intake metabolism, reproductive systems, and immune systems (Li et al., 2021).

6 Such materials can have devastating effects on nature: killing organisms in a lake or river, destroying animals and plants in a contaminated area, causing major reproductive complications in animals, and/or otherwise limiting the ability of an ecosystem to survive (EPA, 2023).

7 Antibiotic residues can be absorbed by plants, interfering with physiological processes and causing potential ecotoxicological effects (Polianciuc et al., 2020).

8 In some cases, severe ecological harms may occur in some locations but not others due to pre-existing background conditions that are beyond the power of the health research actors involved to address. For example, a country’s domestic energy system could make it very difficult for health research projects to operate with minimal carbon emissions, but it would be hard to alter or work around such systems for many (but not all) health research actors. Evidence from the healthcare sector shows how important the broader energy system is to minimising emissions (McGain et al., 2017; Pichler et al., 2019).

9 Cosmopolitan theorists argue that whatever the correct principles of justice are, they ought to apply identically to everyone worldwide (Ypi et al., 2009). They affirm a duty not to harm in ways that deprive people of their right to basic necessities and the right to health extends globally (Pogge, 2002; Shue, 1996). Anti-cosmopolitans or social liberal theorists contend that certain principles of “humanity” may apply identically to everyone worldwide, but principles of “justice” only apply among citizens of the same state (Ypi et al., 2009). They have traditionally relied on the principle of state sovereignty (non-intervention) to limit the harms communities can do to each other to certain forms of military and ideological intervention (Shapcott, 2008). However, some anti-cosmopolitan philosophers purport, where basic rights are being violated or threatened, remedial responsibility may fall on outsiders (see Miller, 2008).
